# Factors associated with choice of biologic among children with Juvenile Idiopathic Arthritis: results from two UK paediatric biologic registers

**DOI:** 10.1093/rheumatology/kev429

**Published:** 2016-01-04

**Authors:** Lianne Kearsley-Fleet, Rebecca Davies, Eileen Baildam, Michael W. Beresford, Helen E. Foster, Taunton R. Southwood, Wendy Thomson, Kimme L. Hyrich

**Affiliations:** ^1^Arthritis Research UK Centre for Epidemiology, Manchester Academic Health Science Centre, The University of Manchester, Manchester,; ^2^Clinical Academic Department of Paediatric Rheumatology,; ^3^Institute of Translational Medicine (Child Health), University of Liverpool, Alder Hey Children’s NHS Foundation Trust, Liverpool,; ^4^Musculoskeletal Research Group, Institute Cellular Medicine, Newcastle University, Newcastle upon Tyne,; ^5^Department of Paediatric Rheumatology, Institute of Child Health, Birmingham Children’s Hospital – NHS Trust and University of Birmingham, Birmingham,; ^6^Arthritis Research UK Centre for Genetics and Genomics, Manchester Academic Health Science Centre and; ^7^NIHR Manchester Musculoskeletal Biomedical Research Unit, Central Manchester University Hospitals NHS Foundation Trust and University of Manchester Partnership, Manchester, UK

**Keywords:** Juvenile Idiopathic Arthritis, epidemiology, biological therapies, information science, attitude of health professionals

## Abstract

**Objective.** The objectives of this study were to describe patients starting first-line biologics for JIA, to describe characteristics over time among patients starting etanercept, and to describe patterns of second biologic prescribing.

**Methods.** The British Society for Paediatric and Adolescent Rheumatology Etanercept Cohort Study, and the Biologics for Children with Rheumatic Diseases study are ongoing prospective observational cohorts, collecting data on patients starting biologic therapy for JIA. Patients registered from 1 January 2010 starting their first biologic were compared between therapies. Patients starting etanercept before 2010 were included to analyse changes in etanercept prescribing. The pathway of patients starting a second biologic was recorded in all patients.

**Results.** To 26 August 2014, 931 patients were recruited starting a first-line biologic (142 Biologics for Children with Rheumatic Diseases; 789 British Society for Paediatric and Adolescent Rheumatology Etanercept Cohort Study). From 2010, patients with systemic JIA (sJIA) were almost exclusively prescribed anakinra or tocilizumab. Choice between anti-TNF therapies was largely driven by history of chronic anterior uveitis (CAU). When investigating trends in patients starting etanercept over time, disease duration at etanercept start, patients with sJIA, a history of CAU, and those who received concomitant oral corticosteroids decreased over time. Patients who started a second biologic from 1 January 2010 showed a similar stratification.

**Conclusion.** Although etanercept remains the most common biologic prescribed for JIA, there has been a clear shift towards the use of alternative biologics, largely driven by disease subtype and history of CAU. This channelling of children towards specific therapies should be considered carefully in future studies and in clinical guidelines and ongoing research.

Rheumatology key messages

- increasingly biologics other than etanercept are being used for JIA as more become available.
- Biologic choice in JIA appears to relate to disease subtype and history of uveitis.
- Channelling towards specific therapies in JIA needs careful consideration in future comparative effectiveness studies.

## Introduction

JIA is the most common chronic rheumatic disease in children and young people (CYP); prevalence in the UK is 1 in 1000 [1]. CYP aged up to 16 years are diagnosed according to the ILAR criteria [2]. In the early 2000s, European licensing of the biologic anti-TNF etanercept revolutionized the management of JIA in CYP with persistent disease who failed to respond to or were intolerant of the traditional non-biologic (nbDMARD) MTX [3].

Since then, a number of other biologic therapies have been approved in Europe for JIA including abatacept, adalimumab, canakinumab and tocilizumab, although in the UK only etanercept and tocilizumab are formally approved by the National Institute for Health and Care Excellence [4, 5]. Furthermore, there is anecdotal evidence that biologics licensed for use in adults with RA, such as other anti-TNF therapies (infliximab), the IL-1 receptor antagonist anakinra and the B-inhibitor rituximab, are also being prescribed in CYP with JIA [6–8]. Etanercept is often the first choice biologic in the treatment of JIA. However, there may be occasions where etanercept is not the preferred choice, for reasons of disease phenotype, effectiveness, safety or clinical context (adherence issues, patient choice). Recent studies have reported that IL-1 and IL-6 inhibiting drugs and IL-1 receptor antagonists, including tocilizumab, canakinumab and anakinra, may be more effective for the treatment of systemic JIA (sJIA) [9–12]. Adalimumab or infliximab may also be the preferred treatment option for CYP with a history of chronic anterior uveitis (CAU), despite a lack of published large head-to-head randomized controlled trials between therapies [13, 14].

Unfortunately, it is also recognized that a proportion of CYP will not respond to their first biologic or will experience adverse effects. There is limited evidence to support the choice of a second or further biologic in these situations, although reports to date suggest ILAR subtype and the availability of other biologics will influence this choice [6]. In one study of patients who initially started etanercept, the majority of patients with sJIA who switched to a second biologic started anakinra, while those without sJIA were more likely to choose a second anti-TNF (adalimumab) [15].

Factors which influence how biologics have been selected in the past, both first-line and on switching, will help inform future clinical practice, guidelines and research. Therefore, the aims of this analysis are to describe disease characteristics among CYP recently starting different first-line biologics for JIA; to describe changes in patient characteristics over time among CYP starting etanercept in light of an expanding evidence base for the efficacy of other biologic therapies for JIA; and to describe patterns of second biologic prescribing among CYP who fail to respond to or are intolerant of their first biologic.

## Methods

### Study setting

This analysis used data collected in two parallel JIA biologic registers. First, the British Society for Paediatric and Adolescent Rheumatology Etanercept Cohort Study (BSPAR-ETN); established in 2004, this study aims to recruit CYP with active JIA at the point of starting etanercept. Second, the Biologics for Children with Rheumatic Diseases Study (BCRD); following recognition of the expanding use of non-etanercept biologics in CYP with JIA, in 2010, a separate national register was established to monitor long-term safety and effectiveness of biologics other than etanercept in CYP with JIA.

BSPAR-ETN was approved by the West Midlands Research Ethics Committee, BCRD was approved by the North West 7 REC Greater Manchester Central Ethics Committee, and written informed consent from all parents (and where appropriate patients) is provided in accordance with the Declaration of Helsinki for both of the studies. We did not require additional ethical approval to analyse data in BSPAR and BCRD.

### Study design

The BSPAR-ETN and BCRD are parallel studies that share the same methodology. Designed as prospective observational cohort studies, they aim to recruit CYP at the point of starting a biologic therapy. Recruitment to these multicentre studies is supported by the National Institute for Health Research Clinical Research Network in England and respective Networks across the devolved nations of the UK. However they do not represent all CYP in the UK on these drugs as registration was not mandatory (although encouraged) and it also did not include children receiving biologic therapies within concurrent clinical trials. The studies are not limited to first time biologic users and both aim to recruit CYP within 6 months of starting a biologic drug. Children can switch between studies but can be tracked through the two studies by their unique National Health Service number.

### Data collection

Baseline information is collected via questionnaires completed by the treating physician or affiliated clinical research nurse. Data items include patient demographics (age, gender), disease duration, ILAR category, current disease activity captured using the JIA core outcome variables (active joint count, limited joint count, ESR, CRP, physician’s global assessment of disease, patient’s global assessment of wellbeing, childhood health assessment questionnaire (CHAQ)) [16], pain visual analogue scale (VAS), history of CAU, details of current and past anti-rheumatic therapies including any prior biologics, and all currently prescribed medications. Follow-up data are collected at 6 months, 1 year and annually thereafter. Information on disease activity measures and changes in drug therapy are included.

### Data analysis

This analysis was divided into three main parts. First, to compare patient characteristics between CYP starting different first-line biologic therapies, we included all patients registered on a first biologic therapy in BSPAR-ETN and BCRD from 1 January 2010, the date on which data collection across all biologics commenced. Baseline characteristics were described and comparisons between all these groups were made using non-parametric descriptive statistics, namely chi-squared and Kruskal–Wallis. Analysis was limited to drugs where at least five CYP had started the therapy.

Second, in order to understand whether there have been changes in patient characteristics over time among CYP at the start of etanercept as their first-line therapy, all patients registered on etanercept as their first biologic therapy in BSPAR-ETN from 1 January 2003 were included. Patients were separated into three cohorts: recruited before 2006; recruited between 1 January 2006 and 31 December 2009; and recruited from 1 January 2010. Comparisons between these groups were made using logistic and linear regression as appropriate.

Finally, to describe patterns of prescribing of a second biologic in CYP who failed to respond or were intolerant of a first biologic, all patients registered at the point of starting either their first or second biologic therapy from 1 January 2010 in either BSPAR-ETN or BCRD were included. Among those CYP who started a second biologic drug (either at the point of first joining the study or after study registration), the pattern of drug–drug switching was described in sJIA patients, non-sJIA patients with a history of CAU, and non-sJIA patients with no history of CAU. The reason for switching was determined from the reason listed for stopping the preceding biologic, as recorded on the case report form and grouped into inadequate response, adverse events, both inadequate response and adverse events, poor adherence and unknown. All analyses were performed using Stata (StataCorp. 2013. Stata Statistical Software: Release 13; StataCorp LP, College Station, TX, USA).

## Results

### Patients registered on a first biologic from 1 January 2010

From 1 January 2010 to 26 August 2014, 348 CYP were recruited at the point of starting their first biologic therapy; 206 (59%) in BSPAR-ETN and 142 (41%) in BCRD (Table 1). This latter study included 60 adalimumab, 30 infliximab, 35 tocilizumab and 15 anakinra patients. One patient started rituximab and one patient had started abatacept, and they were excluded from the analysis. There was a difference between age and disease duration at registration between the drug cohorts, with patients starting etanercept at an older age (median age 12 years; P < 0.001) and patients starting adalimumab with a longer disease duration (median 3 years; P < 0.001).

**Table 1:** Baseline characteristics of patients treated with a first biologic from 1 January 2010; by drug cohorts

	All patients	Etanercept	Adalimumab	Infliximab	Tocilizumab	Anakinra
n	346	206	60	30	35	15
Female, n (%)	224 (65)	140 (68)	40 (67)	17 (57)	16 (46)	11 (73)
Age at registration***, median (IQR), years	11 (6–14)	12 (8–14)	10 (6–14)	8 (5–11)	8 (4–11)	3 (2–13)
Disease duration***, median (IQR), years	2 (1–5)	2 (1–5)	3 (2–6)	2 (2–5)	1 (1–2)	0 (0–1)
	(n = 326)	(n = 193)	(n = 57)	(n = 28)	(n = 34)	(n = 14)
ILAR subtype***, n (%)						
Systemic arthritis	54 (16)	7 (3)	1 (2)	1 (3)	30 (86)	15 (100)
Oligoarthritis: persistent	40 (12)	16 (8)	15 (25)	9 (30)	0	0
Oligoarthritis: extended	59 (17)	34 (17)	17 (28)	8 (27)	0	0
Polyarthritis: RF negative	102 (29)	80 (39)	10 (17)	9 (30)	3 (9)	0
Polyarthritis: RF positive	28 (8)	25 (12)	3 (5)	0	0	0
Enthesitis related arthritis	20 (6)	11 (5)	7 (12)	2 (7)	0	0
Psarthritis	20 (6)	13 (6)	6 (10)	1 (3)	0	0
Undifferentiated arthritis	8 (2)	7 (3%)	0	0	1 (3)	0
Not recorded	15 (4)	13 (6)	1 (2)	0	1 (3)	0
Concomitant MTX***, n (%)	205 (59)	95 (46)	41 (68)	26 (87)	31 (89)	12 (80)
Concomitant oral corticosteroids***, n (%)	61 (18)	17 (8)	8 (13)	6 (20)	23 (66)	7 (47)
Ever had CAU***, n (%)	74 (21)	10 (5)	42 (70)	22 (73)	0	0
Ever had CAU, with no active joints***, n (%)	29 (8)	2 (1)	17 (28)	10 (33)	0	0
Ever had MAS***, n (%)	11 (6)	0	1 (3)	0	5 (17)	5 (42)
	(n = 191)	(n = 88)	(n = 38)	(n = 21)	(n = 30)	(n = 12)
Disease activity, median (IQR)						
Active joint count***	3 (1–7)	4 (1–8)	2 (0–4)	2 (0–6)	3 (0–5)	5 (2–12)
	(n = 310)	(n = 187)	(n = 55)	(n = 27)	(n = 30)	(n = 11)
Limited joint count*	2 (0–5)	3 (1–6)	2 (0–3)	1 (0–6)	2 (0–3)	3 (0–13)
	(n = 302)	(n = 182)	(n = 55)	(n = 24)	(n = 30)	(n = 11)
Physician’s global assessment, 10 cm VAS	3 (1–5)	3 (1–5)	3 (1–4)	2 (1–4)	3 (1–5)	4 (2–6)
	(n = 226)	(n = 136)	(n = 44)	(n = 18)	(n = 20)	(n = 8)
Parent’s global assessment of wellbeing, 10 cm VAS	4 (1–6)	4 (1–6)	3 (1–6)	3 (1–5)	4 (1–8)	5 (4–5)
	(n = 237)	(n = 143)	(n = 45)	(n = 17)	(n = 22)	(n = 10)
Childhood heath assessment questionnaire*, 0–3	0.9 (0.3–1.5)	0.9 (0.3–1.5)	0.8 (0.0–1.4)	0.5 (0.3–1.0)	1.1 (0.4–2.0)	1.6 (1.0–2.1)
	(n = 236)	(n = 147)	(n = 41)	(n = 19)	(n = 22)	(n = 7)
Pain, 10 cm VAS	4 (1–7)	4 (1–7)	3 (1–7)	4 (2–5)	3 (1–6)	6 (4–6)
	(n = 224)	(n = 134)	(n = 43)	(n = 17)	(n = 21)	(n = 9)
ESR**, mm/h	10 (5–26)	10 (5–24)	7 (5–26)	7 (3–14)	24 (5–50)	58 (8–96)
	(n = 282)	(n = 162)	(n = 50)	(n = 29)	(n = 31)	(n = 10)
CRP***, mg/l	5 (3–17)	5 (3–11)	4 (2–6)	4 (1–5)	22 (4–63)	64 (21–99)
	(n = 271)	(n = 145)	(n = 50)	(n = 30)	(n = 34)	(n = 12)
Juvenile arthritis disease activity score, JADAS-71	12 (6–20)	13 (6–19)	10 (6–17)	7 (3–13)	17 (1–22)	23 (7–30)
	(n = 175)	(n = 108)	(n = 33)	(n = 15)	(n = 13)	(n = 6)

Drug cohorts with five or more patients in are represented in this table.

*P < 0.05.

**P < 0.01.

***P < 0.001.

n: number of patients with data available; IQR: interquartile range; CAU: chronic anterior uveitis; MAS: macrophage activation syndrome; VAS, visual analogue scale.

A number of differences were observed between CYP starting different first-line biologics. The majority of children prescribed either tocilizumab (86%) or anakinra (100%) had sJIA compared with <5% of patients starting one of the three anti-TNF therapies (Table 1). Anakinra and tocilizumab were also more likely to be taken in combination with corticosteroids. Children starting anakinra were considerably younger (median age 3 years) compared with those starting tocilizumab (median age 8 years); however, this was not statistically significant (P = 0.308). Patients starting anakinra were also more likely to have a history of macrophage activation syndrome (MAS) (P < 0.001).

There were notable differences between the three anti-TNF therapies, with 70% of children starting adalimumab and 73% of children starting infliximab having a history of CAU, compared with only 5% of children starting etanercept (P < 0.001). There were differences in the ILAR subtypes across these three drugs, with a higher proportion of children with oligoarthritis starting one of the two monoclonal antibodies (adalimumab or infliximab; P < 0.001), although the oligoarthritis subgroup did not all have a history of CAU. Not unexpectedly, the median active and limited joint counts were also lower in children starting adalimumab or infliximab (P < 0.05). A majority of children started their biologic drug in combination with methotrexate (59% overall), although this proportion was lowest for etanercept (46%) and highest for infliximab (87%) and tocilizumab (89%) (P < 0.001). Twenty-nine (8%) patients were reported to have started a biologic with no active joints but with a history of CAU, all of whom started adalimumab (n = 17), infliximab (n = 10) or etanercept (n = 2).

### Comparison of baseline characteristics between patients treated with first-line etanercept over time

In total, 789 CYP were recruited starting etanercept as a first biologic: 231 prior to 2006, 352 between 2006 and 2010, and 206 from 2010 onwards. Disease duration reduced over time (4 years vs 3 years vs 2 years; P < 0.001). In addition, disease activity measures decreased over time, indicated by lower scores across most of the JIA core outcome variables (Table 2). The proportion of CYP starting etanercept in combination with methotrexate reduced over time (57% vs 55% vs 46%; P = 0.029), as well as the proportion of CYP receiving concomitant oral corticosteroids (29% vs 22% vs 8%; P < 0.001). The proportion of children starting etanercept with either sJIA or CAU reduced over time: 16% vs 9% vs 3% (P < 0.001) and 14% vs 6% vs 5% (P < 0.001), respectively.

**Table 2:** Baseline characteristics of first-line etanercept: pre-2006, from 2006 to 2010 and from 2010 onwards

	Etanercept pre-2006	Etanercept 1 January 2006 to 31/12/2009	Etanercept 1 January 2010 onwards	P-values
All patients, n (%)	231 (29)	352 (45)	206 (26)	
Female, n (%)	145 (63)	239 (68)	140 (68)	0.240
Age at registration, median (IQR), years	12 (9–14)	11 (7–14)	12 (8–14)	0.667
Disease duration, median (IQR), years	4 (2–7)	3 (2–6)	2 (1–5)	<0.001
	(n = 227)	(n = 340)	(n = 193)	
ILAR subtype, n (%)				
Systemic arthritis	37 (16)	33 (9)	7 (3)	
Oligoarticular: persistent	4 (2)	11 (3)	16 (8)	
Oligoarticular: extended	43 (19)	60 (17)	34 (17)	
Polyarticular: RF negative	71 (31)	125 (36)	80 (39)	
Polyarticular: RF positive	17 (7)	40 (11)	25 (12)	
Enthesitis related arthritis	19 (8)	31 (9)	11 (5)	
PsA	16 (7)	28 (8)	13 (6)	
Undifferentiated arthritis	22 (10)	17 (5)	7 (3)	
Not recorded	2 (1)	7 (2)	13 (6)	
Concomitant MTX, n (%)	131 (57)	192 (55)	95 (46)	0.029
Concomitant oral corticosteroids, n (%)	67 (29)	79 (22)	17 (8)	<0.001
Ever had chronic anterior uveitis, n (%)	33 (14)	21 (6)	10 (5)	<0.001
Disease activity, median (IQR)				
Active joint count	6 (3**–**12)	5 (2**–**9)	4 (1**–**8)	0.002
	(n = 208)	(n = 315)	(n = 187)	
Limited joint count	6 (3**–**11)	4 (2**–**8)	3 (1**–**6)	<0.001
	(n = 198)	(n = 303)	(n = 182)	
Physician’s global assessment, 10 cm VAS	4 (3**–**6)	4 (2**–**5)	3 (1**–**5)	<0.001
	(n = 158)	(n = 218)	(n = 136)	
Parent’s global assessment of wellbeing, 10 cm VAS	5 (3**–**7)	4 (2**–**7)	4 (1**–**6)	0.003
	(n = 156)	(n = 250)	(n = 143)	
Childhood heath assessment questionnaire, 0–3	1.4 (0.9**–**2.1)	0.9 (0.4**–**1.6)	0.9 (0.3**–**1.5)	<0.001
	(n = 99)	(n = 199)	(n = 147)	
Pain, 10 cm VAS	5 (3**–**7)	4 (1**–**7)	4 (1**–**7)	0.035
	(n = 133)	(n = 232)	(n = 134)	
ESR, mm/h	20 (7**–**46)	16 (6**–**35)	10 (5**–**24)	0.001
	(n = 175)	(n = 284)	(n = 162)	
CRP, mg/l	11 (5**–**38)	7 (4**–**34)	5 (3**–**11)	<0.001
	(n = 178)	(n = 272)	(n = 145)	
Juvenile arthritis disease activity score, JADAS-71	17 (12**–**25)	15 (8**–**21)	13 (6**–**19)	0.003
	(n = 104)	(n = 169)	(n = 108)	

n: number of patients with data available; IQR: interquartile range; VAS, visual analogue scale.

### Patterns of switching from first- to second-line biologics

**Fig. 1:**
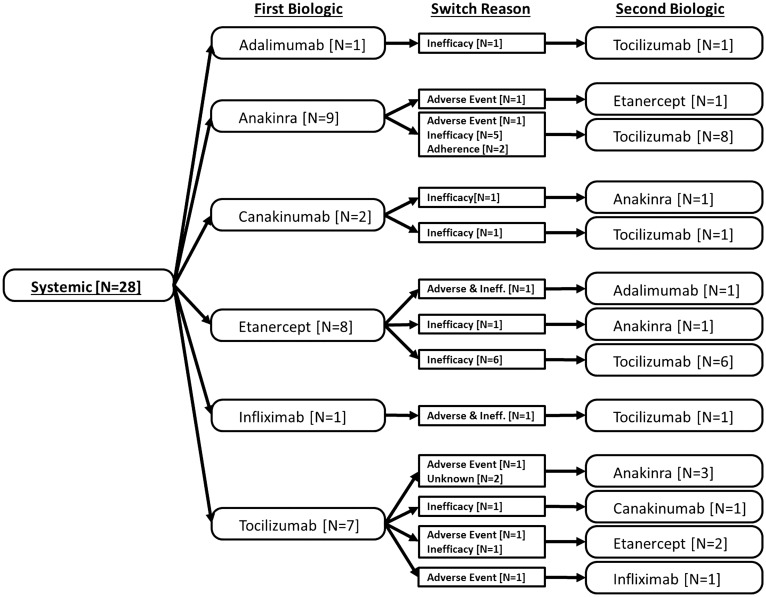
Systemic JIA patients starting second biologic from 2010; pathway from first to second biologic, including reason for switching Pathway from first biologic to second biology. Numbers of first biologic patients are different from Table 1 as this figure includes prior biologic history among those who joined the study at the point of starting a second biologic.

**Fig. 2:**
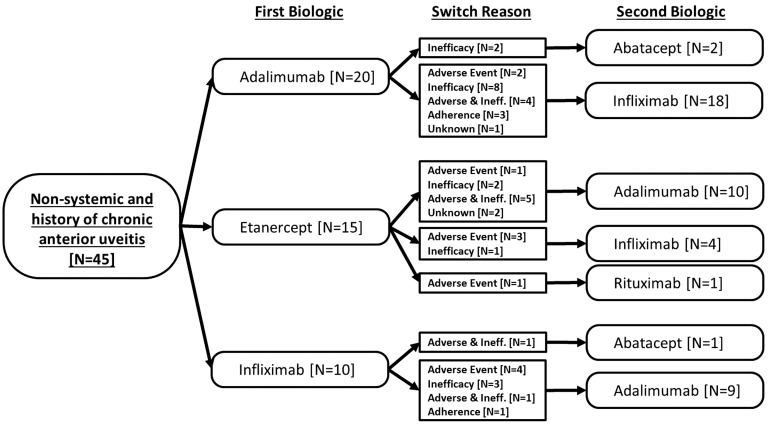
Non-systemic JIA patients with a history of chronic anterior uveitis starting second biologic from 2010, including reason for switching Pathway from first biologic to second biology. Numbers of first biologic patients are different from Table 1 as this figure includes prior biologic history among those who joined the study at the point of starting a second biologic.

**Fig. 3:**
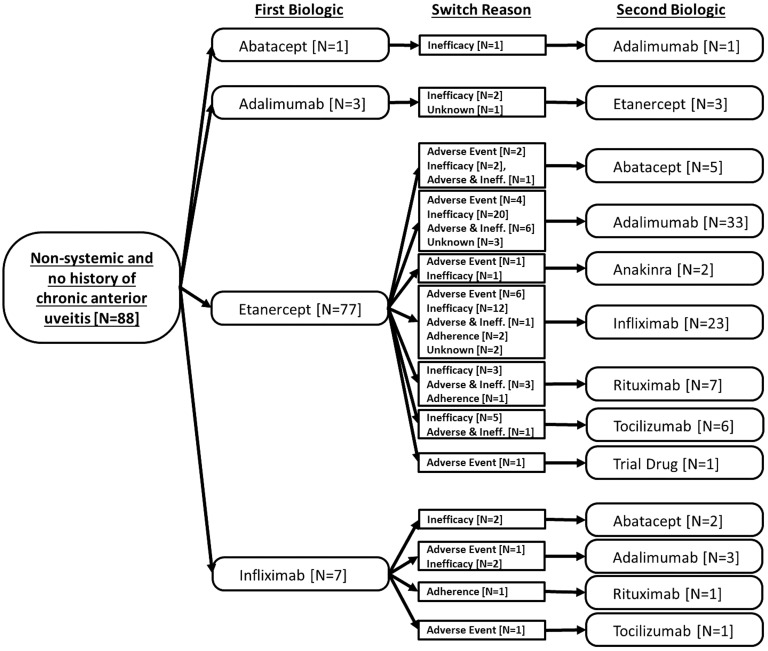
Non-systemic JIA patients with no history of chronic anterior uveitis starting second biologic from 2010, including reason for switching Pathway from first biologic to second biology. Numbers of first biologic patients are different from Table 1 as this figure includes prior biologic history among those who joined the study at the point of starting a second biologic.

From 1 January 2010, a total of 115 CYP were recruited at the point of starting a second biologic. In addition, of the 348 CYP registered on a first-line biologic, 46 subsequently started a second biologic over a median of 2.2 years of follow-up, providing a total of 161 patients switching to a second biologic. The reasons for switching were inadequate response in 86 patients (53%), adverse events in 32 (20%), both inadequate response and adverse events in 22 (14%), issues with adherence in 9 (6%) and unknown in 12 (7%). Figures 1–3 show the pattern of switching between first and second biologics.

Among patients with sJIA (Fig. 1) who started a second biologic (n = 28), the majority of patients switched to tocilizumab (17 patients). Five patients started anakinra and five patients started an anti-TNF therapy. Among the seven patients whose first drug was tocilizumab, three switched to an anti-TNF therapy and four switched to an agent which inhibits IL-1.

Forty-five patients with a history of CAU started a second biologic over the study period. The majority of patients either switched to or switched between adalimumab and infliximab. No patient switched to etanercept (Fig. 2).

Finally, among those patients with non-systemic JIA and no history of CAU (n = 88), a majority switched to a second anti-TNF therapy (n = 63) (the vast majority had received etanercept as their first biologic), although there were also children who switched to abatacept, tocilizumab, anakinra and rituximab (Fig. 3).

## Discussion

This study has shown that the spectrum of biologics being used in JIA since 2010 is wide, with five main biologics being prescribed; etanercept, adalimumab, infliximab, tocilizumab and anakinra. There was apparent channelling of CYP towards different first-line therapies in association with disease phenotype, namely ILAR category (sJIA vs non-sJIA) and the presence of CAU. A similar pattern was also seen in CYP switching to a second biologic.

Since 2010, a majority of children in our study starting tocilizumab or anakinra had sJIA, which follows recent evidence of efficacy of these drugs for CYP with sJIA [9, 10, 17] and data showing that etanercept may be less effective for sJIA compared with other subtypes [18]. The median age of children starting anakinra was considerably younger and this may reflect the different modes of delivery of these two drugs over the study period (subcutaneous anakinra vs intravenous tocilizumab), which may have influenced patient or parent choice. Patients starting anakinra were also more likely to have a history of MAS. MAS is a complication of rheumatic diseases and has been more frequently associated with sJIA [19]. Various case reports have indicated that patients with sJIA-associated MAS have been successfully treated with anakinra [20–22] which may have influenced physician choice.

There was also marked channelling of patients with CAU towards monoclonal antibody TNF inhibitors and away from etanercept. This is consistent with the Dutch national Arthritis and Biologics in Children Register, where 4% of etanercept patients and 71% of adalimumab patients had a history of CAU at baseline [23]. Infliximab has been reported to be efficacious in the treatment of JIA-CAU [24, 25], and to be superior to etanercept in small studies with limited patient numbers [26, 27]. There have also been reports that etanercept therapy is no more beneficial than placebo in the treatment of CAU [28] and further registry data have shown that a history of CAU was associated with etanercept discontinuation [29]. It has also been reported that disease activity [30] and CAU inflammation [31] are improved in CYP with JIA-CAU treated with adalimumab. However, overall the evidence is very limited in small patient numbers [32], with no account for differences in patient characteristics between the compared cohorts [33, 34]. Taken together, these data may explain why infliximab or adalimumab was chosen over etanercept in patients with a history of CAU. Controlled trials investigating the best drug for JIA-CAU are ongoing [35] and may provide more accurate evidence for the use of certain biologics in these patients [32]. Most specifically, there are currently two ongoing randomized controlled trials investigating adalimumab in combination with MTX ADUVITE and SYCAMORE trials) in the treatment of JIA-CAU [13, 36].

This study has also demonstrated that since its introduction, etanercept is now being prescribed earlier in disease in children with lower levels of disease activity and severity. There has also been a reduction in the proportion of CYP with sJIA or a history of CAU starting etanercept, which was evident even in the years prior to 2010, suggesting that a widening choice of biologics was already affecting drug selection. This is consistent with studies in the Dutch Arthritis and Biologics in Children register [6] and the German Biologics JIA Registry [37], as well as in line with recent EULAR recommendations for RA, where earlier treatment with anti-TNF inhibitors in patients who have failed to reach low disease activity with nbDMARDs is recommended [38].

In CYP switching to a second biologic, similar channelling to that seen with the choice of a first biologic was also seen. The majority of CYP with sJIA switched to tocilizumab and anakinra. However, in those patients who started tocilizumab first-line, pattern of switching was less clear, indicating a lack of information regarding the best choice of therapy when tocilizumab fails. The majority of patients without sJIA but with a history of CAU switched to adalimumab or infliximab, with no patients switching to etanercept. Patients without sJIA and with no history of CAU tended to switch to a second anti-TNF therapy. Overall, etanercept was less frequently seen as a second biologic, possibly because it is the only biologic approved by National Institute for Health and Care Excellence for first line therapy in polyarticular JIA and therefore more commonly used in this position [4]. In addition, both adalimumab and infliximab are commonly used anti-TNF therapies, and despite infliximab not being licensed in children with JIA, both are commonly used in paediatric rheumatology practice [6]. Across all children, the most common reason for switching was inadequate response to their first biologic.

This analysis was completed in a large number of CYP with JIA in paediatric rheumatology centres across the UK. Many drug cohorts were included and contributed a greater understanding of biologic prescribing patterns in recent years. In addition, due to the continued availability of etanercept patients in BSPAR-ETN from the beginning of the 21^st^ century, differences in patient characteristics could be compared with more recent patients.

As with most observational studies, the data are not without their limitations. These registers are observational studies where registration into a biologic cohort is encouraged but not mandatory. Therefore, the data cannot be used to draw conclusions about the relative proportions of patients prescribed different biologic drugs in the UK. The dataset is not exhaustive and therefore the data should only be used to comment on observed patient differences. There may be further unmeasured factors that also influenced choice of therapies. As there was no qualitative analysis of patient or physician choice, we can only assume that these observed differences are associated with patient and physician choice, but cannot be certain. While the aim of both the registers was to recruit CYP with active JIA, some patients who started first-line biologic had no active joints, but a history of CAU. All of these patients started either adalimumab or infliximab, with a few starting etanercept, which therefore suggests that in a proportion of CYP, it is their eye disease rather than their joint disease driving the decision to start a biologic.

Many CYP with JIA are now receiving biologic therapies in the UK. Although etanercept appears to be the most common biologic prescribed for JIA, there has been a clear shift towards the use of alternative biologics, including unlicensed biologics, in certain patients, largely driven by disease subtype (sJIA vs non-sJIA) and the presence of CAU. This channelling of certain children towards specific therapies will need to be considered both in terms of future comparative effectiveness studies and also as a guide to ongoing research priorities within rheumatology. Whilst trends of patient characteristics over time have been previously described in both an etanercept registry and a register comparing etanercept vs adalimumab, this is the first description of biological therapy prescribing patterns in CYP with JIA in all of the biological therapies currently available in the UK. As both of the UK paediatric studies continue to develop, future analyses investigating patient outcomes in the various drug cohorts will be possible, as well as addressing the question of the best choice of biologic therapy as first-line use for which limited clinical experience exists.
